# Systemic analysis shows that cold exposure modulates triglyceride accumulation and phospholipid distribution in mice

**DOI:** 10.1371/journal.pone.0313205

**Published:** 2024-11-07

**Authors:** Isabella James, Raghav Jain, Gina Wade, Philip C. Stevenson, Albert Koulman, Judith Simcox, Samuel Furse

**Affiliations:** 1 Department of Biochemistry, College of Agriculture and Life Sciences, University of Wisconsin-Madison, Madison, Wisconsin, United States of America; 2 Biological Chemistry Group, Royal Botanic Gardens Kew, Richmond, Surrey, United Kingdom; 3 Natural Resources Institute, University of Greenwich, Chatham, Kent, United Kingdom; 4 Core Metabolomics and Lipidomics Laboratory, Wellcome-MRC Institute of Metabolic Science, University of Cambridge, Cambridge, United Kingdom; 5 Institute of Metabolic Science-Metabolic Research Laboratories and Medical Research Council Metabolic Diseases Unit, University of Cambridge, Cambridge, United Kingdom; Louisiana State University Health Sciences Center, UNITED STATES OF AMERICA

## Abstract

Environmental exposure to cold is increasingly being associated with changes in metabolism. We developed and tested the hypothesis that exposure to cold drives systemic effects in lipid metabolism. Specifically, (i) that energy storage and provision adapts to the cold by altering triglyceride distribution and (ii) that membranes adapt to cold conditions by becoming more unsaturated. These hypotheses were designed to identify the underlying mechanisms that govern the response of mammalian systems to cold. To test these hypotheses, we used a metabolic network analysis. An established model of cold exposure was used, from which lipidomics data that represents the system was collected. The network analysis showed that triglyceride metabolism is altered on exposure to cold, with several smaller effects that are not straightforward, such as changes to the abundance and distribution of odd chain fatty acids. The range and profile of phosphatidylcholine and phosphatidylinositol were modified, but there was little change in phosphatidylethanolamine or sphingomyelin. These results support the hypothesis, and show that exposure to cold is a system-wide phenomenon that requires or drives changes across a range of metabolic pathways.

## Introduction

The effects of environmental stresses on development and health have been a topic of ongoing interest for decades. However, precisely what mechanisms are altered by environmental stresses such as temperature changes are not always easy to determine. This is partly because a variety of pathways can be affected by a single stress and a single pathway can be affected by a number of stresses. It is also because several, related pathways may be affected in unpredictable ways. One of the major adaptations to the cold is lipid remodelling [1–4]. Lipid remodelling is complicated to understand because lipids have a range of roles *in vivo*; fatty-acid-containing metabolites (lipids) include species with roles in energy storage and distribution (triglycerides) and species with physical roles (phospholipids). Studies of cold exposure in mammals has shown greater levels of desaturation in membrane lipids in lipids found in the circulation [5, 6]. This is consistent with our understanding of lipid-water systems in which greater unsaturation is associated with greater fluidity [7, 8], suggesting that lipids are being made more unsaturated so that membranes can remain fluid despite a drop in temperature.

However, unsaturation is not a useful adaptation for managing energy storage and distribution in the cold, *i*.*e*. in triglyceride metabolism. Cold exposure is exceptionally energetically demanding, with endotherms in particular requiring a large increase in energy expenditure to maintain optimum temperature for biochemical reactions [9]. In wild mammals living in temperate zones, cold exposure is managed through a combination of increased dietary intake in the autumn to fuel heat-releasing tissues such as brown adipose tissue (BAT) during the winter. These observations inspired early work into how lipid metabolism was modified on acclimatisation to cold conditions [10], with early studies showing dramatic differences in saponified hepatic lipids [11, 12]. Further studies expanded upon these observations to show increased lipid desaturation in individual tissues, including studies on adipose [13–16], skeletal muscle [17], liver [18] and even one study on several tissues from the same individual animals [19].

The changes in gross lipid composition in individual tissues led recently to a more focused investigation of effects across tissues. Jain *et al*. investigated the lipid composition of a number of tissues in mice exposed to cold. They found evidence for an orchestrated lipid remodelling across tissues and circulating lipids [4]. Specifically, Jain *et al*. found that that the liver and brown adipose tissue were major contributors and consumers of acyl carnitines and that the supply of ceramides in the circulation was altered on exposure to cold. These results suggest that there are whole-system effects of environmental stresses such as cold. To date, whole-system effects have not been investigated, only work on individual tissues have been reported. This is particularly important in the context of lipid metabolism as lipid metabolism is well known to be a whole-system phenomenon [20–24]. Jain *et al*.’s study showed that cold exposure drives changes to energy distribution (through mobilisation of fatty acids from TGs) and changes to structure (ceramides in the circulation), hinting that there are whole-system effects of cold exposure.

If these results are looked at in the context of whole-system effects, possible mechanisms can be explored. Specifically, the system could adapt to colder conditions by using triglycerides to release more heat and to change the structural lipids (membrane components) to maintain membrane function at lower temperatures. Changes to the supply of ceramides suggests that sphingolipid metabolism may be altered in response to colder conditions. Another mechanism centres on unsaturation of phospholipids; lipids with more unsaturated bonds are more fluid than those with fewer [7, 8], suggesting that the effects of cold could be resisted by altering membrane structure.

**Fig 1:**
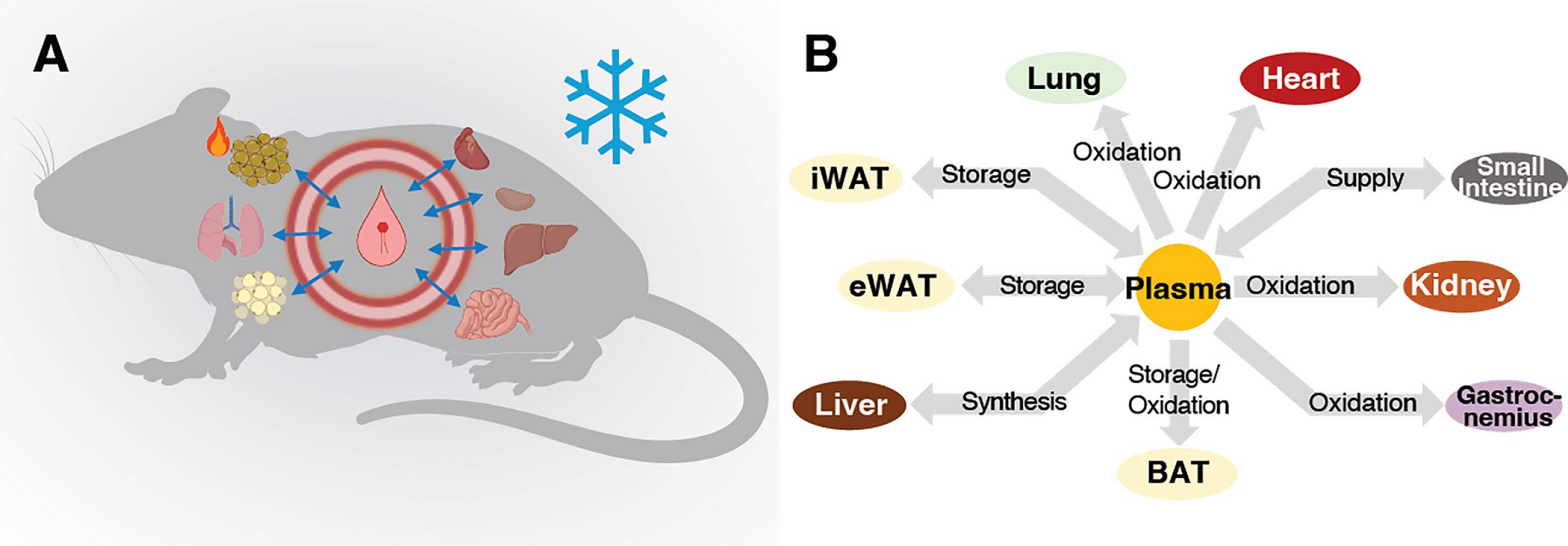
The mouse model of cold exposure used in the present study. Panel **A**, schematic picture of the model used; **B**, the compartments used to describe the metabolic network of the mice of the mouse model. Connections are based on known metabolic interactions between compartments.

The present study is focused on identifying responses and thus possible mechanisms for acclimatisation to cold. A deeper understanding of the coordinated lipid response and inter-organ cross talk is needed. We interpreted this as a need for a network or systemic analysis. The evidence accumulated to date led us to the hypothesis that exposure to cold conditions drives alterations in the supply of energy (triglycerides, mobilised from stores) and the physical structure of membranes (phospholipids, to increase fluidity), along with changes to endogenous lipid biosynthesis (energy storage). To test these hypotheses, we applied Lipid Traffic Analysis [20, 22, 25, 26] to a known mouse model of cold exposure [4] (*Fig 1A*). Traffic Analysis is a relatively new method for testing hypotheses in metabolic systems that uses lipidomics data to plot the distribution of lipids throughout both the control and experimental organisms (systems). This can be either quantitative analyses that use RADAR plots to indicate which tissues show the greatest difference between groups, or Switch Analyses that show how the distribution of lipids differs between groups. Switch Analyses show which lipids are missing or accumulated in different parts of the system. This is important as it shows how the way synthesis, degradation and transport of lipids differs between Control and Experimental systems. The Traffic Analysis plots show this visually, describing the number of variables of each lipid class through the system. LTA plots show immediately which parts of the system and which lipid pathways or classes differ between groups. The parts of the system that contrast the most sharply between groups are referred to as control points. Importantly, control points can only be identified where the whole system is investigated. For example, a lipid that is unique to one compartment can only be identified as such if many other compartments are also profiled. Lipid Traffic Analysis therefore uses the known biology of the system in the analysis to identify how metabolism changes across and in different parts of the system. The biological network for this mouse model is shown in *Fig 1B*, in which all tissues in the periphery are represented. This network was used to identify which lipid pathways were altered, and how and where, following exposure to cold in male mice. We began by testing of the hypothesis that cold exposure leads to gross systemic changes to lipid metabolism.

## Materials and methods

### Animal model

No animal work was done in this project. Some of the data we used came from animal samples, which were obtained in a separate study by Jain *et al*. [4].

### RNA extraction and cDNA synthesis

All steps were performed on ice using treated surfaces and equipment (ethanol 70% and RNAse zap. Tissues were sectioned (~20 mg per sample, 1·5 mL plastic Eppendorf tube) and homogenized (1 mL, cold Trizol [ThermoFisher, cat. no. 15596026]) with a metal bead in a TissueLyzer (30 Hz, 40 s; Qiagen cat. no. 85300). Chloroform (200 μL) was added to each sample before mixing (benchtop vortexer, 30 s) and centrifugation (12k × *g*, 15 min, 4°C). The aqueous phase was removed to a new tube and diluted (IPA, 500 μL, 4°C). This mixture was stored (-80°C, 1-7d). Samples were thawed and centrifuged (12k × *g*, 15 min, 4°C). The supernatant was removed and the pellet washed (70% ethanol, 4°C, 1 mL, twice). The pellet was then dried (30–60 min, stream of nitrogen) and resuspended (ultrapure water [Invitrogen cat. no. 10977023], 20–50 μL), and the concentration was measured (NanoDrop). RNA (500 ng) was added to an RT-PCR master mix prepared according to the kit instructions (High Capacity cDNA Reverse Transcription kit, Thermo cat. no. 46-688-13) to give a consistent final volume (20 μL). The mixture was then thermos-cycled (25°C for 10 min, 37°C for 120 min, 85°C for 5 min, held at 12°C). The cDNA was then diluted (10×) and stored (-20°C) until qPCR commenced.

### qPCR

Samples were pooled and 3 serial dilutions performed. A blank sample of ultrapure water was also run. Master mizes were created for each gene using forward and reverse primer and SYBR green master mix (Thermo cat. no. 25778). cDNA (2 μL) and master mix (5 μL) were added to each well. Data was processed using an in-house R script [4].

### Triglyceride assays

Plasma samples (20 μL) were used untreated. Tissue samples (20–50 mg) were added to a tube with a metal bead, PBS (250 μL), MeOH (225 μL) and TBME (750 μL), and homogenized (TissueLyzer), before being centrifuged (16·1 k × *g*, 15 min, 4°C). The upper layer was removed to a new tube and dried (vacuum centrifuge). The triglycerides were then resuspended (IPA, 225 μL IPA + Triton 25 μL and stored at 4°C). The pellet from the tissue was resuspended (RIPA buffer, 450 μL, containing protease inhibitor) and agitated (4°C, 10 min). The samples were centrifuged to pellet insoluble debris, and the supernatant removed for BCA assays to normalize the TAG assays to total protein. A Wako kit was used for the triglyceride assay. Each sample (4 μL) was injected into a 96-well plate, in triplicate. Reagent 1 was added (90uL/well) and heated (37°C, 5 min) and absorbance measured as a background (600 nm). Reagent 2 (30 μL/well) was then added, the mixture heated (37°C, 5 min) and absorbance measured (600 nm). Sample concentrations were calculated using a standard curve of dilutions of the standard from the kit. Concentrations were normalized to total protein levels from the BCA assay.

### BCA assays

BCA assays were performed using a kit (Thermo cat. no. 23225). Heart samples were either undiluted, 1:10 diluted, and 1:5 diluted protein extracts collected as described above. For FPLC plasma fractions, BCA assays were performed on undiluted, 1:10 diluted, and 1:5 diluted fractions. For unfractionated plasma samples, BCA assays were performed on 1:50 and 1:100 diluted samples.

### Fast Protein Liquid Chromatography (FPLC)

Plasma was collected from fasted mice (6 h, RT or 4°C). Plasma was pooled from several mice (500 μL per group), and then diluted (500 μL, PBS). The diluted plasma (1 mL) was injected onto the FPLC (run in PBS 10 mM, *p*H 7·4, with 0.02% sodium azide, flow rate of 0·5 mL/min). The first 10 mL of flow was discarded, and 1 mL fractions were collected after 20 min. The samples were run on a Superose 6 (cat. no. 17-0537-01) and a Superdex 200 (cat. no. 17-1088-01) column to separate lipoprotein complexes by size. Triglyceride, cholesterol, and BCA assays were run on the fractions to identify when HDLs, LDLs and albumin eluted. For triglyceride and cholesterol assays, the samples (30 μL) were combined with the appropriate reagent (250 μL, Pointe scientific) and heated (37°C, 30 min) before the absorbance was measured (500 nm). Early fractions were treated as background measurements. The background-subtracted absorbances were converted to mg/dL through normalization to a standard curve. The assays were performed in duplicate for each sample.

### Lipid Traffic Analysis

Lipid Traffic Analysis v3.0 was used in the present study [26], recently updated from v2.3 [22, 25, 27, 28].

In LTA v3.0, the calculation of Error Normalised Fold Change (ENFC [20]) was done as previously described, using Eq 1. These are presented as RADAR plots for simplicity.


$$Error\;normalised\;fold\;change=\frac{{log}_{10}(\frac{{\bar{x}}_{E}}{{\bar{x}}_{C}})}{\left(\sqrt{\frac{\left(a^{2}+b^{2}\right)}{2}}\right)}$$ Eq 1

For the network part of the LTA, the Switch Analyses, pie charts of the numbers of variables were generated from the data output from the LTA software (the data used can be found in *Supplementary Materials*). Larger pie charts (on arrows) were generated to represent the number of variables found in pairs of metabolically adjacent compartments (***B***-type variables). Smaller pie charts were generated to represent isolated variables, *i*.*e*. the ones found in only one tissue for that group (***U***-type). The total number of lipid variables of each type was calculated and shown as a table (inset), including the lipids that are found throughout the network (***A***-type variables). In the new version of the LTA software (v3.0), the code for the Switch Analysis was also updated to include alignment of lists in order to produce an additional output describing the traffic of each variable, for targeted analyses [26]. A novel lipid type was added, referred to as ***N***_***2***_***-***type, in addition to the existing ***U***-, ***A***- and ***B***-type lipids. ***N***_***2***_-type lipids are distinct from ***B***-type lipids in that they are found in any two compartments. The full code for Lipid Traffic Analysis v3.0 is available publicly (https://pypi.org/project/lipidta/). Variables were regarded as present if they had a signal strength >0 in ≥66% of samples per group. Total numbers of variables for each class and of each type are shown in *Table 1*. Statistics for interpreting the meaning of these numbers are presented; *J* represents the Jaccard-Tanimoto coefficient for the comparison, with accompanying *p* value, as a measure of the similarity between the variables identified in the two phenotypes for each comparison. The *p* value shown represents the probability that the difference between the lists of variables for the two phenotypes occurred by random chance. In LTA plots of quantitative differences (Error Normalised Fold Change, ENFC [20]) are RADAR plots that show which tissues differ and by what degree. Lipidomics data for the Switch Analyses and Abundance Analysis were generated by Jain *et al*. [4]. Processed lipidomics data are available in the *Supplementary Information* (S1 Data)

**Table 1:** The numbers of variables of *A-*, *B-*, *U-* and *N*_*2*_- lipid types from a Lipid Traffic Analysis (LTA) of a murine model of cold exposure.

Lipid	Total No. variables	*A*-type		*B*-type		*U*-type		*N*_*2*_-type	
		Room temp.	Cold Exposed	Room temp.	Cold Exposed	Room temp.	Cold Exposed	Room temp.	Cold Exposed
AC	19	0	0	4	7	3	4	1	3
PC	308	9	9	112	110	85	94	43	46
PE	143	0	0	25	25	37	41	20	20
PG	79	0	0	4	4	28	30	6	10
PI	73	2	2	22	18	12	14	12	14
PS	77	0	0	4	4	26	27	18	18
SLs	294	4	4	51	51	92	95	83	80
TG	334	34	32	49	50	98	101	37	36

LTA consisted of Control (Room temp.) and Cold Exposed groups of mice. Total number variable indicates the number of lipids identified in that class. ***A***-type lipids are ones found throughout the system, ***B***-type lipids are found in metabolically adjacent compartments (*i*.*e*. compartments between which lipids are passed), ***U***-type lipids are only found in one compartment (but may be in either group) and ***N***_***2***_-type lipids are found in any two compartments. AC, acyl carnitine; PC, phosphatidylcholine, PE, phosphatidylethanolamine; PG, phosphatidylglycerol; PI, phosphatidylinositol; PS, phosphatidylserine; SLs, Sphingolipids (comprising sphingomyelins, ceramides, hexosylceramides, dihydrosphingosine); TG, triglycerides.

### Statistical methods

Univariate and bivariate statistical analyses, and error normalised fold change (ENFC) [20], were calculated in Microsoft Excel 2016. Graphs were prepared in Excel 2016 or OriginLab 2018. Calculations of Jaccard-Tanimoto Coefficients (JTCs, *J*) and associated *p-*values were used as a non-parametric measure of the distinctions between lipid variables associated with phenotype(s) [20]. The *p*-value associated with each *J* represents the probability that the difference between the lists of variables for the two phenotypes occurred by random chance, representing both the number of variables only found in either of the two groups and the order of the binary list and is described in detail by Rahman *et al*. [29]. It is a non-parametric statistic that should not be confused with a Student’s *t-*test. We observe that *p* values below 0.55 are consistent the two groups being compared each having lipid(s) that the other does not. All lipidomic data were assumed to be unequally distributed and heteroscedastic and so the appropriate type of non-parametric test was applied.

## Results

### 1. Hypothesis: Cold exposure drives systemic changes in lipid metabolism

**Fig 2:**
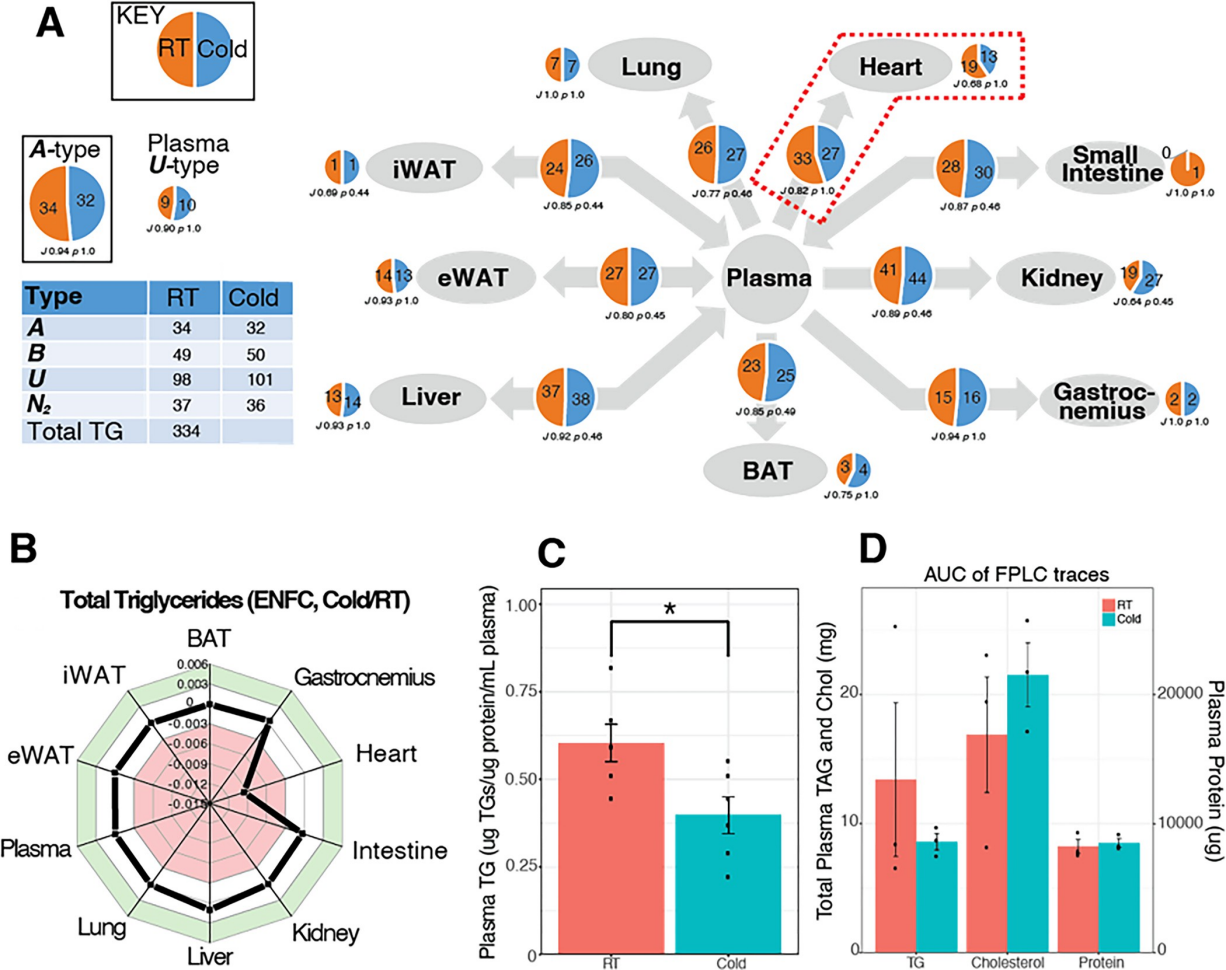
Traffic and plasma analyses of triglycerides from a mouse model of cold exposure. Panel **A**, Traffic Analysis of triglycerides (TG). Larger pie charts (on arrows) represent variables found in the two adjacent compartments (***B***-type variables). Smaller pie charts represent isolated variables (***U***-type). The table (inset) shows the total number of lipid variables of each type for the network. *J* represents the Jaccard-Tanimoto coefficient for the comparison, with accompanying *p* value, as a measure of the similarity between the variables identified in the two phenotypes for each comparison. The *p* value shown represents the probability that the difference between the lists of variables for the two phenotypes occurred by random chance. TGs include all adducts of whole TGs and the DGs arising from in-source fragmentation of TGs during data collection. Panel **B**, Error-normalised fold change (ENFC [20]) for the total abundance of TG, between cold-exposed and control mice. Panel **C**, Total TG in plasma in the two groups (μg/mL plasma), *p* <0.05. Panel **D**, Area Under the Curve (AUC) calculations for the Fast Protein Liquid Chromatography (FPLC).

We began by testing the hypothesis that there are systemic changes in the traffic of lipids associated with energy distribution such as triglycerides (TGs), and lipids with structural roles (phospholipids). Lipid Traffic Analyses (LTA, v3.0 [26]) showed that the metabolism of triglycerides (*Fig 2A*), acyl carnitines (*S2A Fig*) and several phospholipid classes (*e*.*g*. PC, *S2B Fig*; PI, *S2C Fig*) were altered by exposure to cold. LTA of TGs showed that the number of ***A-***type TGs falls on exposure to cold, losing TG(52:6) and TG(51:3). This suggests that both polyunsaturated and odd-chain fatty acids (PUFA and OCFA, respectively) are involved in the metabolic changes associated with exposure to cold across the whole system. There is also evidence for shifts within sections of the system. Traffic Analysis also showed that the profile of lipids across several tissues, rather than the absolute number, differed through the system (***B-*** and ***U-***type lipids, *Fig 2A*). There was evidence for changes to the traffic to all adipose tissues, liver, lung, kidney, and small intestine as both the number of lipids differed as did the profile (*J* < 1, *p* < 0·55). The heart differed from this. In that compartment, the number of variables was lower after exposure to cold, with six fewer ***B-***type TG variables and six fewer ***U-***type variables, and the total TG was much less than for other tissues (*Fig 2B*). Absolute measurements showed the TG pool was at least 50% smaller in the hearts of mice exposed to cold (*S1A and S1B Fig*). The total mass of TGs *in circulo* was around 30% less in the cold-exposed group (*Fig 2C;* full FPLC trace shown in *S1C Fig*), with the concentration of circulating TG also slightly lower in the group exposed to cold (*Fig 2D*). These results highlight the challenges of capturing a dynamic system with single tissue lipid measurements. Triglycerides are an energy rich fuel source, and their levels indicate both production and consumption by various tissues. LTA is able to overcome these challenges by assessing inter-organ cross talk.

The LTA of TGs therefore showed that the routing of TGs through the system was altered by exposure to cold conditions in male mice, with control points in several organs that have different roles. The LTA of acyl carnitines (ACs, *S2A Fig*) is consistent with this, showing a greater range of ACs through the system in mice exposed to cold. These results showed that there were system-wide effects in fatty acid metabolism, hinting that there may also be changes to the metabolism of structural lipids. These too were investigated through two major classes, phosphatidylcholine and phosphatidylinositol.

The LTA of phosphatidylcholine (PC) showed that the number of ***B-***type variables differed little between the two groups (typically <5%) and the number of shared variables between each comparison (the overlap between groups) was >0.8 (*J* value). However, the (non-parametric) *p* values show that for most of the system were <0.55. This is consistent with both groups containing lipid(s) that the other does not. This shows that on exposure to cold, some lipids were lost and others were gained. In short, mice exposed to cold have a similar number, but different profile, of PCs (*S2B Fig*). The shifts in the profile were generally lower than for TGs, at 10% or less. In general, the differences in profile were driven by PCs containing docosahexaenoic acid (FA(22:6), DHA) and docosapentaenoic acid (FA(22:5), DPA). PCs containing DHA were more prevalent in control mice, whereas PCs containing DPA were more prevalent in cold-exposed mice. Notably, there was evidence for changes in lipid traffic to two adipose depots, inguinal white adipose tissue (iWAT) and BAT. Evidence for changes to the phospholipid composition of adipose suggests there is a change in the membrane composition of these tissues, driven by cold. The number of ***U****-*type PC variables was larger in mice exposed to cold in several compartments, including heart, lung, BAT and liver, with a smaller number in one (small intestine) and a reconfiguration in another (gastrocnemius). These were not generally polyunsaturated fatty acid-containing PCs, so the local reorganisation of PCs may not be driven by increases in fluidising lipids.

The LTA of phosphatidylcholine (*S2B Fig*) showed clear and system-wide changes in lipid metabolism, and thus supports the hypothesis. However, it does not show a trend for an increase in fluidity as this is typically associated with shorter chains and/or greater unsaturation [7, 8]. We therefore tested the same hypothesis by doing a Traffic Analysis of phosphatidylinositol (PI), an important membrane component that can have dramatic effects on lipid-water systems [30, 31]. The modulation to PI traffic driven by exposure to cold led to a smaller number of PIs, though virtually no change in the profile (*S2C Fig*). The trend was typically that PI(18:1/20:3) and PI(18:1/20:5) were not found in animals exposed to cold. Once again, this shows that there was a change to phospholipid metabolism that suggests structural change to lipid membranes but this does not appear to be consistent with an increase in membrane fluidity.

Changes to the distribution of TGs, acyl carnitines, PCs and PIs, and with different changes to the different pathways and different control points, showed that exposure to cold drove changes to the routing of lipid traffic in several ways. Furthermore, some fatty acids disappeared from PC and PIs without appearing in others. This suggests that exposure to cold drives distinct effects in different classes and also that the supply and demand of fatty acids changes. Importantly, polyunsaturated fatty acids expected to increase fluidity were lost rather than gained in PC and PI, suggesting that fluidity has not increased in membranes. This strongly suggests that there exposure to cold conditions drives both general and specific systemic changes in lipid metabolism. We therefore looked for quantitative changes to known endogenous lipid synthesis pathways.

### 2. Hypothesis: Cold exposure drives changes to endogenous lipid biosynthesis

**Fig 3:**
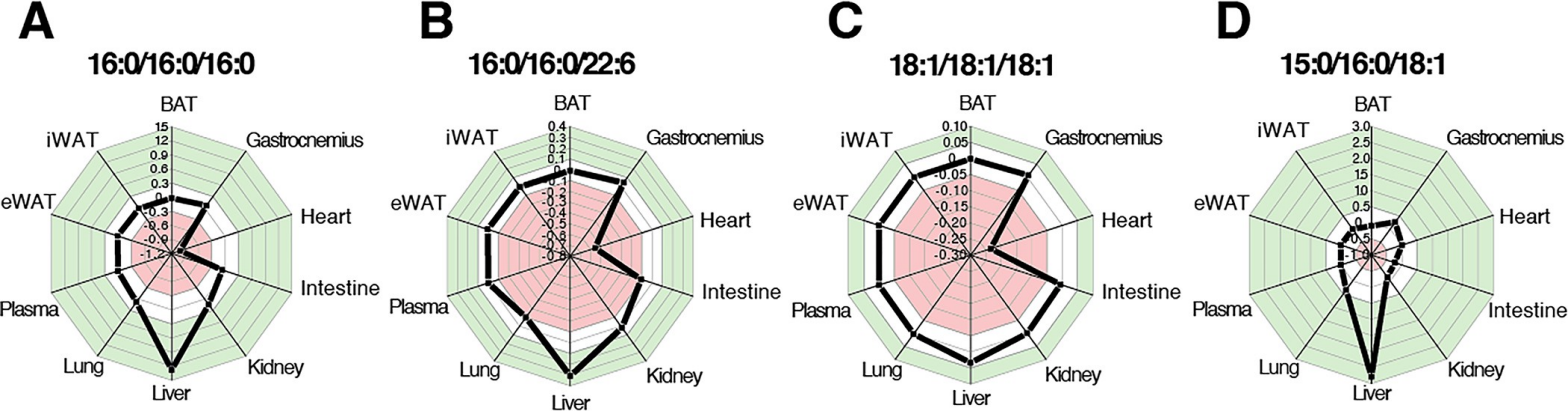
Error-normalised fold change plots of lipid biomarkers of *de novo* lipogenesis. Panel **A**, TG(16:0/16:0/16:0); **B**, TG(16:0/16:0/22:6); **C**, TG(18:2/18:2/18:2); **D**, TG(15:0/16:0/18:1). These plots show the ratio of the experimental group divided by the control group, scaled by the error (standard deviation) of the measurements taken [20].

An Abundance Analysis of TGs associated with *de novo* lipogenesis [32], such as TG(16:0/16:0/16:0), PUFA-containing TGs, *e*.*g*. TG(16:0/16:0/22:6), and other abundant TGs found in the circulation of mammals, *e*.*g*. TG(18:1/18:1/18:1) was done to test this hypothesis (*Fig 3A–3C*, additional examples *S3–S5 Figs*). The error-normalised fold change (ENFC, Abundance Analysis [20]) of most individual TG isoforms reflected the overall trend for TGs after exposure to cold; a much lower abundance in the heart, a slight increase in the liver and only minor changes elsewhere. A notable exception was TG(18:2/18:2/18:2) that showed considerable increase in the liver, lesser increases in the gastrocnemius, kidney and lung, and no change in the heart (*S5C Fig*). TGs comprising the most abundant OCFA isoforms, FA(15:0) and FA(17:0), conformed to the general trend. These are typically the result of modification of fatty acids modified by the product of the gene *hacl1*. The two most abundant TGs comprising FA(17:0) and FA(17:1) showed a similar pattern to total TGs (*S6A–S6C Fig*). However, the expression of *hacl1* itself fell (*S6D Fig*), suggesting several changes in biological infrastructure that controls the biosynthesis and distribution of OCFA-containing species on exposure to cold. The traffic of 15-carbon fatty acids also shows a different but also complicated pattern. TG(15:0/16:0/18:1) and TG(15:0/16:0/18:2) appear in several tissues but only of systems exposed to cold, suggesting that they are either made separately in several compartments or made in one compartment and distributed. The Abundance Analyses showed that there was a considerable increase of the abundance of TG(15:0/16:0/18:1) and TG(15:0/16:0/18:2) in liver (*Fig 3D and S7A Fig*) with small changes elsewhere. TG(15:0/18:1/18:2) and TG(15:0/18:2/18:2) (*S7B and S7C Fig* respectively) accumulate in the lung, and TG(15:0/16:0/18:2) and TG(15:0/18:1/18:2) showed accumulation in the gastrocnemius. This pattern is somewhat consistent with TG(18:2/18:2/18:2), that was more abundant in the gastrocnemius, liver and lung, however both differ from the typical pattern for TGs. It is surprising that FA(15:0)-containing TGs should appear or increase in abundance after exposure to cold. Mammalian fatty acid synthase is only capable of synthesizing even chain fatty acids, and odd chain fatty acids are typically associated with the dietary intake or production by the gut microbiota; FA(15:0) is specifically associated with consumption of dairy fat [33].

These results are remarkable because they show that there is very precise control of both fatty acid and TG metabolism on exposure to cold, and that TGs associated with *de novo* lipogenesis (DNL) were not mobilised to oxidative tissues as part of the adaptation to cold exposure. Instead, exogenously produced TGs such as TG(18:2/18:2/18:2) were accumulated to the heart and gastrocnemius. Importantly, virtually all fats were accumulated in the liver in animals that had been exposed to cold, consistent with other studies [34]. As the metabolic turnover is higher in animals on exposure to cold [6, 35, 36], we suggest that the control points that describe the change to TG metabolism after exposure to cold conditions are in the liver (poorer transfer of TGs to the circulation) and in the heart (poorer uptake from the circulation).

The patterns observed in PC are broadly reflected in the Abundance Analysis of TGs. Abundant FA(16:0)-containing PCs are typically less concentrated in the hearts of mice exposed to cold, although all are more abundant in the epididymal white adipose tissue (eWAT) of cold-exposed mice (*S8 Fig*). The abundance of FA(15:0)- and FA(17:0)-containing PCs is more varied, with many being more abundant in the livers of cold-exposed mice. PC(15:0/18:2) and PC(17:0/18:2) were notable exceptions as they were more abundant in the hearts of mice exposed to cold, which is not a pattern observed in odd-chain-containing TGs or TG(18:2/18:2/18:2). Several FA(17:0)-containing PCs were more abundant in the livers of mice exposed to cold conditions (*S9 Fig*), however several mechanisms may be responsible for this. One possibility is that the gene *hacl1* is expressed more rapidly in the liver, or the proteins degraded less quickly, on exposure to cold.

### 3. Hypothesis: Disappearance of essential, PUFA-containing TGs and PCs is driven by exposure to cold

**Fig 4:**
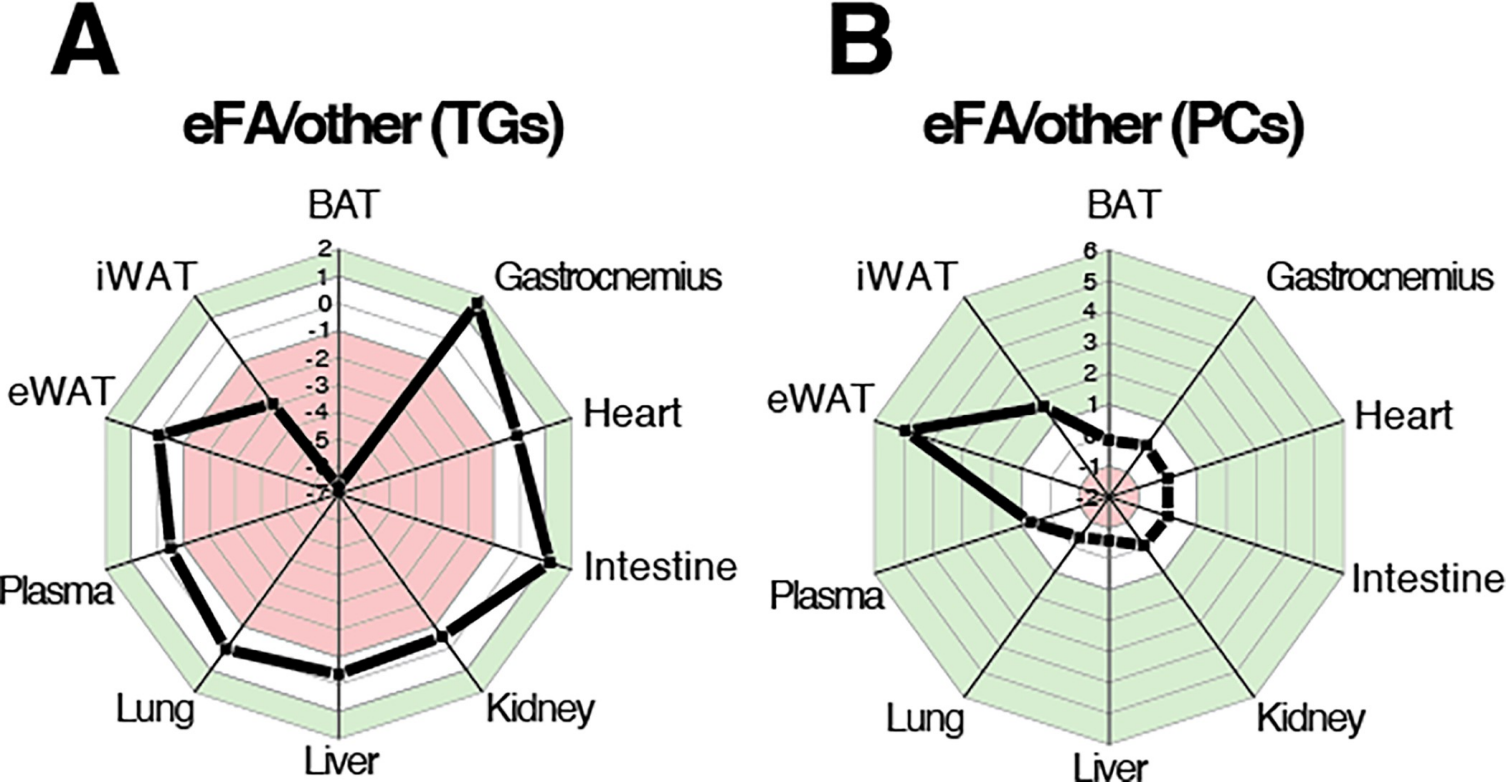
Error-normalised fold change plots of the ratio of essential polyunsaturated fatty acids against all other isoforms. Panel A, TGs; B, PCs. These plots show the value for the ratio of experimental group divided by the control group, scaled by the error (standard deviation) of the measurements taken [20].

The ENFC of the ratio of lipids comprising essential polyunsaturated fatty acids such as DHA and DPA to all others in the same class are shown in *Fig 4*. This shows that essential PUFA-containing TG and PC were altered by exposure to cold, but in different ways. Essential PUFAs were removed from TGs in the BAT on exposure to cold but were accumulated in PCs in the eWAT and to an extent the iWAT. There was little or no change to PUFA-containing PC or TG in the heart and liver. To understand the effect better, we looked at the contributions of individual PUFAs. The most abundant TGs containing FA(22:6) and FA(22:5) show lower abundance in the heart and the same or higher in the livers of cold-exposed mice, with FA(22:5)-PCs higher in the gastrocnemius (*S4 Fig*). In adipose tissues, the abundance of these lipids was the same or slightly lower. This indicated that the supply of PUFAs was altered but not in the way anticipated; if our hypothesis was correct, one would expect PUFAs to be accumulated in phospholipids rather than in triglycerides in muscle tissue. In fact FA(22:5)-containing PCs were accumulated in several different tissues after exposure to cold, including at least small increases in the liver and gastrocnemius (*S10 Fig*). There was also evidence for a general increase in FA(22:6)-containing PCs in the eWAT with a modest increase in iWAT. There was a consistent loss of PUFA-containing PCs from lung tissue.

Measurement of total saturated, *mono*-unsaturated and poly-unsaturated fatty acids (SFA, MUFA and PUFA) in plasma shows minimal difference in the supply of any of these types of fatty acid. There were pronounced differences in the fatty acid composition of lipids in the livers of the cold-exposed and control groups and a trend towards increase of SFA, MUFA and PUFAs in the eWAT, but no large-scale changes (*S11 Fig*).

## Discussion

In this paper, we have shown that the systemic control of lipid metabolism differs in male mice after they were exposed to cold. A number of orthogonal pathways were modulated: triglycerides, phosphatidylcholine, phosphatidylinositol, acyl carnitines, endogenous fatty acid biosynthesis and chain shortening. Network analysis also showed the distribution of lipids through the system, identifying which lipids were associated with what parts of the system, and revealing particular effects in heart and liver. This study follows investigations into the metabolic consequences of exposure to cold going back as far as the 1950s but provides new insights using systemic analyses supported by other measurements.

An early study into how lipid metabolism was affected by exposure to cold conditions showed that the ratio of solid to liquid fatty acids (a proxy for saturated and unsaturated fatty acids) was dependent on temperature [11]. Specifically, the ratio of liquid to solid fatty acid did not change from 2°C to 24°C in rats, but that the ratio increases at 35°C through an increase in the abundance of solid fatty acids. The results from the present study showed that there was no appreciable increase in polyunsaturated lipids in mice exposed to cold, which is consistent with past work. The present study also shows that the shifts within individual lipids therefore reflect the overall trend for fatty acids. Other observations are also reflected in the present results; the slight increase in the abundance of most triglycerides in the liver in the present study reflects earlier work that found a faster rate of beta-oxidation in rats exposed to cold [18]. This study found that some triglyceride isoforms did not conform to this trend, suggesting that triglycerides are sorted or otherwise filtered for different roles after exposure to cold.

Specifically, this was observed in FA(18:2)-containing TGs, where both TG(15:0/18:2/18:2) and TG(18:2/18:2/18:2) were handled differently to TGs more generally but also differently to each other. This suggests that TGs are ‘read’ carefully, not only when they contain FA(18:2) but also when they contain odd chain FAs. A recent study of skeletal muscle from pigs found that animals exposed to cold had more FA(15:0) and FA(18:2) [17], consistent with the greater abundance of FA(15:0)- and FA(18:2)-containing lipids seen here in mice, however whole-system analysis has yet to be done in pigs for a full comparison between species.

Importantly, local effects are only part of the story. Single tissues do not act in isolation, they are dependent on the supply of lipids from the circulation and also contribute to it, changing the supply to other tissues. Thus, single-tissue studies cannot explain how the system adapts to an environmental stress. This and the evidence that several tissues respond to cold conditions led us to undertake network analyses on a mouse model of this exposure to cold. However, network analyses of metabolomics data are relatively rare and have yet been used in studies of exposure to cold. To date, network analyses such as Traffic Analysis have been applied to nutritional programming [20, 28], diabetes [21, 25, 27], and the dietary intake of fatty acids [22]. These results show that a physical environmental stress has system-wide effects of similar breadth to both dietary change and the metabolic dysregulation associated with gestational diabetes. The systemic effects of dietary interventions on mammals have also been investigated using flux quantification methods, using molecular labelling and mathematical approaches to calculate the cycling of circulating nutrients [37].

A similar ‘fluxomics’ approach may also be useful in studies of cold exposure to understand the cross-talk between pathways, or incorporation of multiple time points of cold exposure with Traffic Analysis. In the present study, there was evidence that mice exposed to cold lost PCs comprising FA(22:6) and gained PCs comprising FA(22:5). FA(22:5) is not known to be made in mammals by saturation of FA(22:6), one potential source is removal of FA(22:6) and replacement with FA(22:5) either from stores or produced *de novo* using ELOVL2/5. Traffic Analyses of male mice supplemented with FA(22:6) showed that it was associated with lower activity of ELOVL2/5 in vastus muscle, *i*.*e*. slower conversion of FA(20:5) to FA(22:5) [22]. One possibility is that the removal of FA(22:6) after cold exposure may drive the synthesis of FA(22:5). If true, this might provide an explanation for the absence of FA(20:5)-containing PIs from cold-exposed mice: the FA(20:5) in PI may be used to biosynthesise FA(22:5) that is then used to produce PC. In this study, FA(22:6)-containing lipids did not increase or accumulate in any tissue measured on exposure to cold. As this study was focused on the periphery, we suggest the hypothesis that exposure to cold could lead to greater traffic of FA(22:6)-containing lipids to the central nervous system. In that case, the expectation would be that the lipid vector for FA(22:6) would be a *lyso*-PC as LPC(22:6) is known to be transported into the CNS through major facilitator superfamily domain-containing protein 2 (MFSD2A) [38, 39].

Notably, there are therapeutic benefits of cold exposure in the periphery, particularly for diabetes [40–42]. Changes to lipid metabolism have been suggested to be responsible for disruption of insulin sensitivity in a mouse model of increased risk of T2DM after gestational diabetes. In that hypothesis, the stiffening of membranes, driven by an increase in saturated fatty acids from *de novo* lipogenesis, was suggested to be responsible for changes to the shape and thus reduction in activity of insulin receptors [27]. The consumption of triglycerides associated with exposure to cold in the present study is consistent with the reduction in adiposity seen in clinical interventions of cold therapy. Thus the consumption of FA(16:0) may be responsible for the changes observed. This may also explain the observations of increased single-carbon transfer on exposure to cold, noted in this study of mice but also another study on pigs [17]. Shorter chains are known to reduce melting temperature of lipid-water systems [7, 8] however it is not clear why shorter chains are preferred over desaturation. One possibility is that desaturation produces too strong an effect in a system of which most cells are not directly exposed to the lower air temperature. These results raise the question of what the source of FA(15:0) is. FA(15:0) is usually understood to come from dairy fat, whereas FA(17:0) is expected to come from activity of the product of *hacl1*. However, these mice were not fed any dairy products at all. This heavily implies that under cold stress at least, FA(16:0) can be shortened by one carbon *in vivo*.

## Conclusion

Our study shows that and environmental stress such as exposure to cold has several whole-system effects. Some of these are general whereas others are more specific to particular parts of the system. There were contrasting effects between pathways, however all pathways were affected to some degree. There was little evidence for increased fluidity of membranes driven by increased unsaturation of phospholipids. The evidence for several, specific, system-wide effects shows that cold has been a selection pressure on mammals and so the evidence gathered reveals part of a sophisticated adaptation to an environmental stress. The results of the present study provide an explanation for the efficacy of the therapeutic use of cold in treating hyperlipidaemia, which is associated with atherosclerosis and cardiovascular disease. How this treatment compares to other interventions for hyperlipidaemia are not yet known. One would expect cold, exercise and dietary changes to place different demands on lipid metabolism. It is not yet clear which or which combination of these would provide the ideal therapeutic combination to treat hyperlipidaemia, however a mechanism-based investigation of all three may provide sufficient detail about them to enable combinations to be tailored to the needs of individual patients.

## Supporting information

S1 Data Measured abundance of lipids from all samples. (CSV)

S1 Fig Abundance of triglycerides in heart tissue and fractionation of plasma. Panel **A**, Concentration of triglycerides in heart determined by colorimetric assay; **B**, Concentration of triglycerides determined by mass spectrometry; **C**, Fractionation of plasma using FPLC, measuring triglyceride and plasma protein concentration.
(DOCX)

S2 Fig Traffic Analyses of major lipid classes from a mouse model of cold exposure. Panel **A**, Traffic Analysis of acyl carnitines; **B**, Traffic Analysis of phosphatidylcholines (PC); **C**, Traffic Analysis of phosphatidylinositols (PI). Larger pie charts (on arrows) represent variables found in the two adjacent compartments (***B***-type variables). Smaller pie charts represent isolated variables (***U***-type). The table (inset) shows the total number of lipid variables of each type for the network. *J* is the Jaccard-Tanimoto coefficient for the comparison, with accompanying *p* value, as a measure of the similarity between the lists of variables for each comparison. The *p* value shown represents the probability that the difference between the lists of variables for the two phenotypes occurred by random chance.
(DOCX)

S3 Fig Error-normalised fold change plots of triglyceride biomarkers of *de novo* lipogenesis. Panel **A**, TG(14:0/16:0/18:1); **B**, TG(16:0/16:0/18:0); **C**, TG(16:0/16:0/18:1). These plots show the ratio of the experimental group divided by the control group, scaled by the error (standard deviation) of the measurements taken [20].
(DOCX)

S4 Fig Error-normalised fold change plots of triglycerides comprising poly-unsaturated fatty acids. Panel **A**, TG(18:2/18:2/22:6); **B**, TG(18:0/18:1/22:6); **C**, TG(18:1/18:1/22:6); **D**, TG(18:2/18:2/22:5); **E**, TG(16:0/16:0/22:6). These plots show the ratio of the experimental group divided by the control group, scaled by the error (standard deviation) of the measurements taken [20].
(DOCX)

S5 Fig Error-normalised fold change plots of triglycerides comprising fatty acids expected to be drawn from dietary intake. Panel **A**, TG(18:1/18:1/18:1); **B**, TG(54:5); **C**, TG(18:2/18:2/18:2). These plots show the ratio of the experimental group divided by the control group, scaled by the error (standard deviation) of the measurements taken [20].
(DOCX)

S6 Fig Error-normalised fold change plots of triglycerides comprising fatty acids expected to be shortened endogenously, by the product of *hacl1*, and expression of the gene. Panel **A**, TG(17:0/18:1/18:2); **B**, TG(17:1/18:1/18:2); **C**, TG(16:0/17:0/18:1); **D**, Normalized expression of *hacl1*. These plots show the ratio of the experimental group divided by the control group, scaled by the error (standard deviation) of the measurements taken [20].
(DOCX)

S7 Fig Error-normalised fold change plots of triglycerides comprising pentadecanoic acid. Panel **A**, TG(15:0/16:0/18:2); **B**, TG(15:0/18:1/18:2); **C**, TG(15:0/18:2/18:2). These plots show the ratio of the experimental group divided by the control group, scaled by the error (standard deviation) of the measurements taken [20].
(DOCX)

S8 Fig Error-normalised fold change plots of abundant isoforms of phosphatidylcholine that comprise palmitic and stearic acids. These plots show the ratio of the experimental group divided by the control group, scaled by the error (standard deviation) of the measurements taken [20].
(DOCX)

S9 Fig Error-normalised fold change plots of odd-chain-containing phosphatidylcholines. Top row, FA(15:0)-containing fatty acids; bottom row, FA(17:0)-containing fatty acids. These plots show the ratio of the experimental group divided by the control group, scaled by the error (standard deviation) of the measurements taken [20].
(DOCX)

S10 Fig Error-normalised fold change plots of polyunsaturated-fatty-acid-containing phosphatidylcholines. Top row, FA(22:5)-containing PCs; bottom row, FA(22:6)-containing PCs. These plots show the ratio of the experimental group divided by the control group, scaled by the error (standard deviation) of the measurements taken [20].
(DOCX)

S11 Fig Concentration of fatty acids from all lipids, grouped into saturated (SFA), mono-unsaturated (MUFA) and poly-unsaturated (PUFA). Panel **A**, Lipids from plasma; **B**, Lipids from the liver; **C,** Lipids from eWAT.
(DOCX)
