# Supplementary material for: Systemic analysis shows that cold exposure modulates triglyceride accumulation and phospholipid distribution in mice
Source: PLoS One. 2024 Nov 7;19(11):e0313205. doi: 10.1371/journal.pone.0313205 (PMC11542792; doi:10.1371/journal.pone.0313205)
Supplement: S1 Fig — Panel A, Concentration of triglycerides in heart determined by colorimetric assay; B, Concentration of triglycerides determined by mass spectrometry; C, Fractionation of plasma using FPLC, measuring triglyceride and plasma protein concentration. (DOCX) [file pone.0313205.s002.docx]

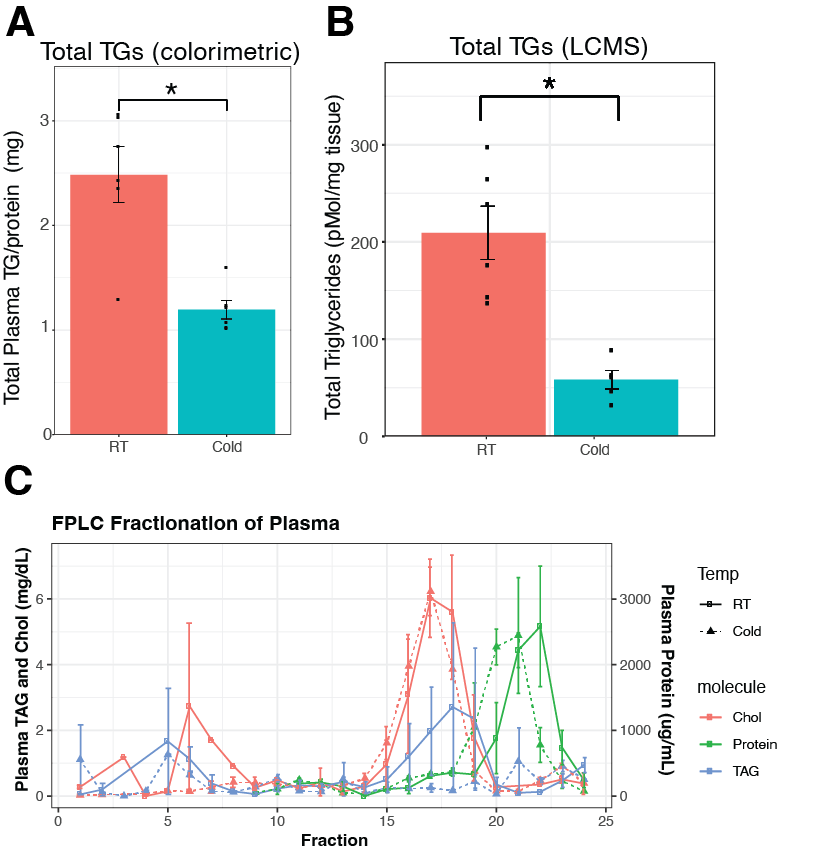


**Fig. S1. Abundance of triglycerides in heart tissue and fractionation of plasma.** Panel **A**, Concentration of triglycerides in heart determined by colorimetric assay; **B**, Concentration of triglycerides determined by mass spectrometry; **C**, Fractionation of plasma using FPLC, measuring triglyceride and plasma protein concentration.
