# Supplementary material for: Systemic analysis shows that cold exposure modulates triglyceride accumulation and phospholipid distribution in mice
Source: PLoS One. 2024 Nov 7;19(11):e0313205. doi: 10.1371/journal.pone.0313205 (PMC11542792; doi:10.1371/journal.pone.0313205)
Supplement: S2 Fig — Panel A, Traffic Analysis of acyl carnitines; B, Traffic Analysis of phosphatidylcholines (PC); C, Traffic Analysis of phosphatidylinositols (PI). Larger pie charts (on arrows) represent variables found in the two adjacent compartments (B-type variables). Smaller pie charts represent isolated variables (U-type). The table (inset) shows the total number of lipid variables of each type for the network. J is the Jaccard-Tanimoto coefficient for the comparison, with accompanying p value, as a measure of the similarity between the lists of variables for each comparison. The p value shown represents the probability that the difference between the lists of variables for the two phenotypes occurred by random chance. (DOCX) [file pone.0313205.s003.docx]

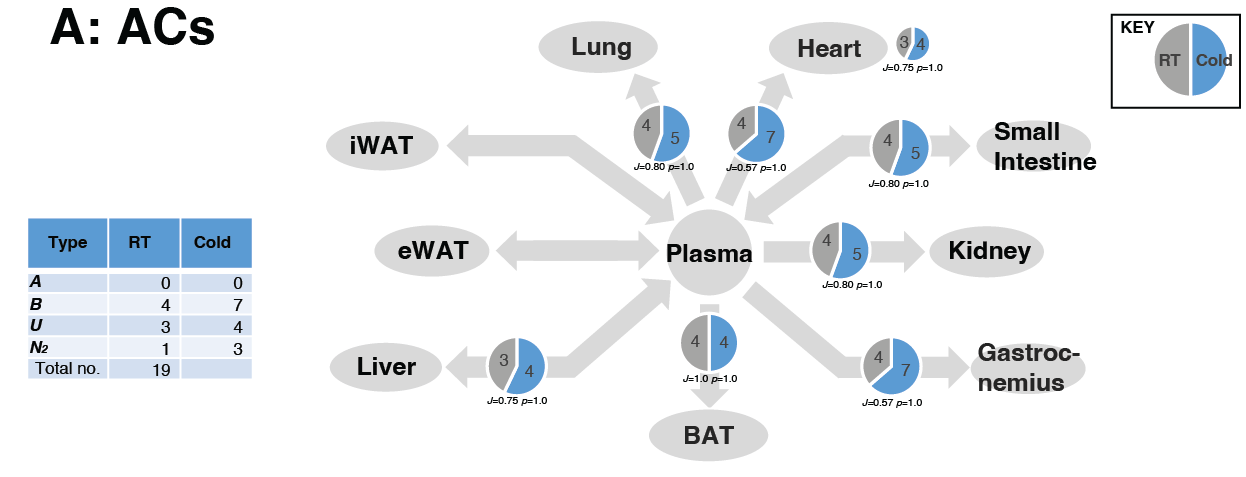


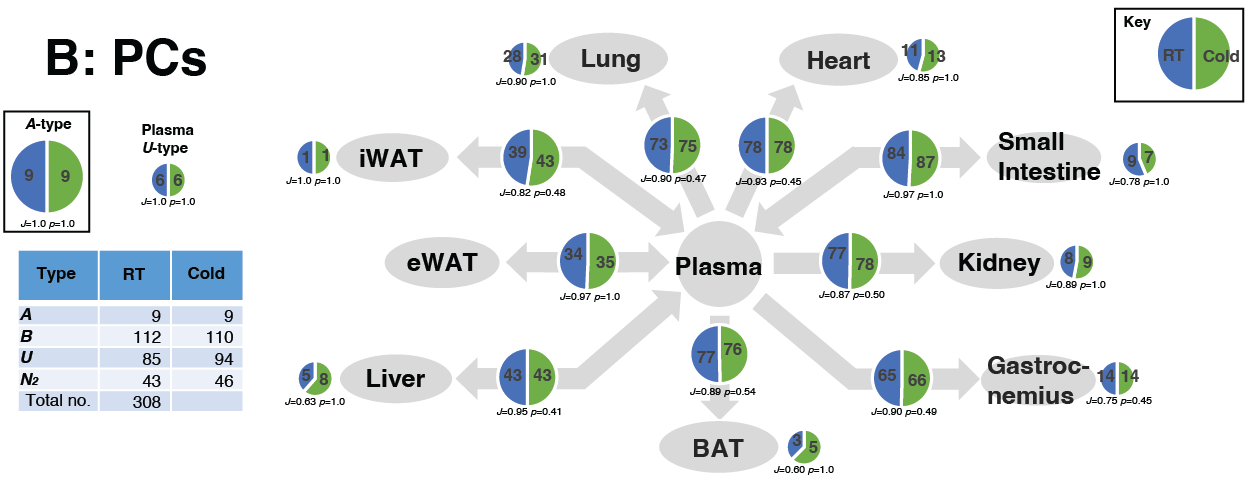


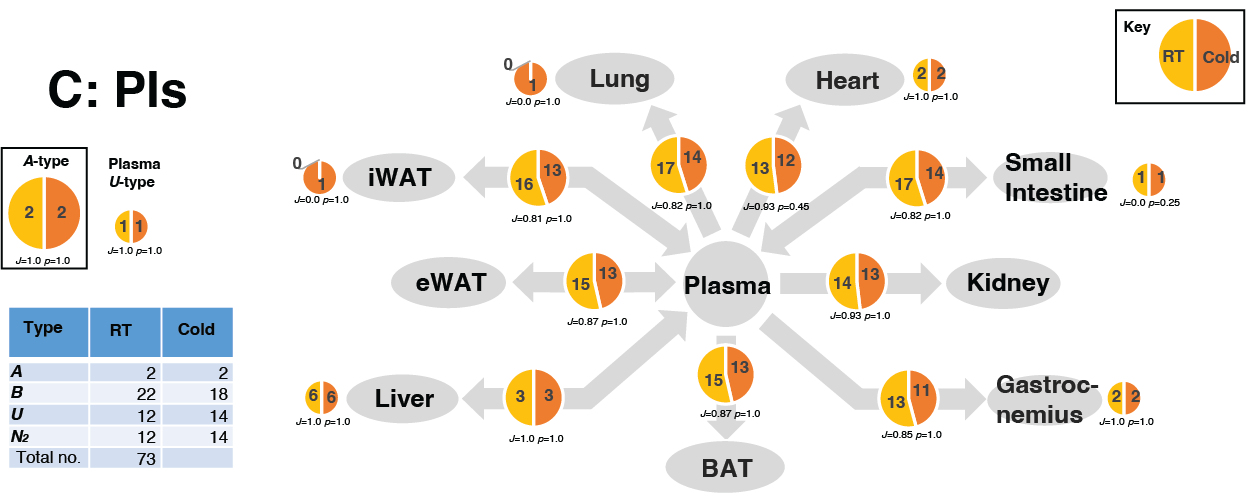


**Fig. S2. Traffic Analyses of major lipid classes from a mouse model of cold exposure.** Panel **A**, Traffic Analysis of acyl carnitines; **B**, Traffic Analysis of phosphatidylcholines (PC); **C**, Traffic Analysis of phosphatidylinositols (PI). Larger pie charts (on arrows) represent variables found in the two adjacent compartments (***B***-type variables). Smaller pie charts represent isolated variables (***U***-type). The table (inset) shows the total number of lipid variables of each type for the network. *J* is the Jaccard-Tanimoto coefficient for the comparison, with accompanying *p* value, as a measure of the similarity between the lists of variables for each comparison. The *p* value shown represents the probability that the difference between the lists of variables for the two phenotypes occurred by random chance.
