# Supplementary material for: Systemic analysis shows that cold exposure modulates triglyceride accumulation and phospholipid distribution in mice
Source: PLoS One. 2024 Nov 7;19(11):e0313205. doi: 10.1371/journal.pone.0313205 (PMC11542792; doi:10.1371/journal.pone.0313205)
Supplement: S3 Fig — Panel A, TG(14:0/16:0/18:1); B, TG(16:0/16:0/18:0); C, TG(16:0/16:0/18:1). These plots show the ratio of the experimental group divided by the control group, scaled by the error (standard deviation) of the measurements taken [20]. (DOCX) [file pone.0313205.s004.docx]

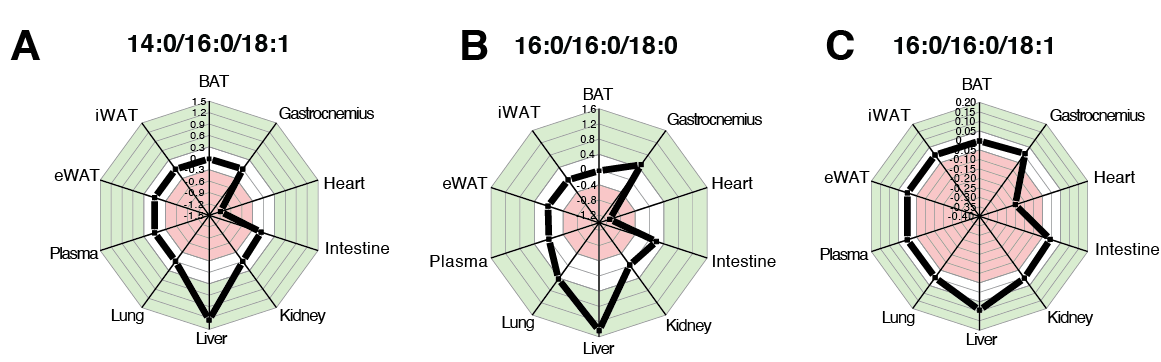


**Fig. S3. Error-normalised fold change plots of triglyceride biomarkers of *de novo* lipogenesis.** Panel **A**, TG(14:0/16:0/18:1); **B**, TG(16:0/16:0/18:0); **C**, TG(16:0/16:0/18:1). These plots show the ratio of the experimental group divided by the control group, scaled by the error (standard deviation) of the measurements taken[1].
