# Supplementary material for: Systemic analysis shows that cold exposure modulates triglyceride accumulation and phospholipid distribution in mice
Source: PLoS One. 2024 Nov 7;19(11):e0313205. doi: 10.1371/journal.pone.0313205 (PMC11542792; doi:10.1371/journal.pone.0313205)
Supplement: S4 Fig — Panel A, TG(18:2/18:2/22:6); B, TG(18:0/18:1/22:6); C, TG(18:1/18:1/22:6); D, TG(18:2/18:2/22:5); E, TG(16:0/16:0/22:6). These plots show the ratio of the experimental group divided by the control group, scaled by the error (standard deviation) of the measurements taken [20]. (DOCX) [file pone.0313205.s005.docx]

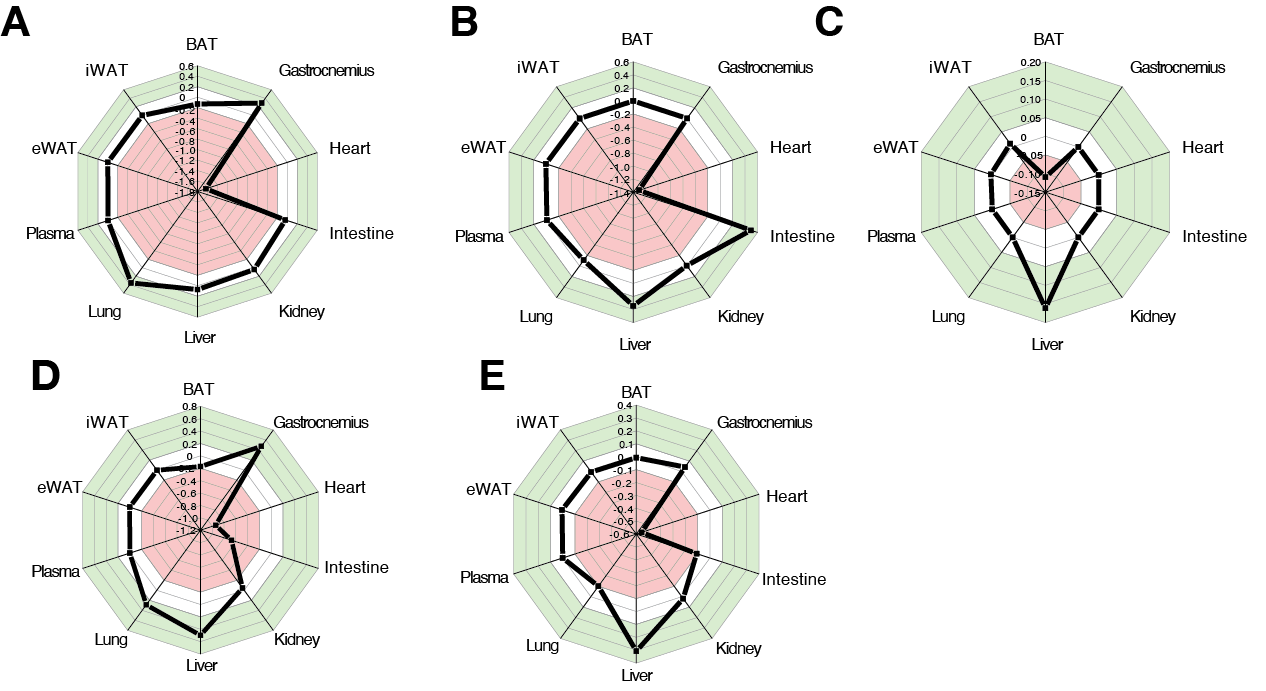


**Fig. S4. Error-normalised fold change plots of triglycerides comprising poly-unsaturated fatty acids.** Panel **A**, TG(18:2/18:2/22:6); **B**, TG(18:0/18:1/22:6); **C**, TG(18:1/18:1/22:6); **D**, TG(18:2/18:2/22:5); **E**, TG(16:0/16:0/22:6). These plots show the ratio of the experimental group divided by the control group, scaled by the error (standard deviation) of the measurements taken[1].
