# Supplementary material for: Systemic analysis shows that cold exposure modulates triglyceride accumulation and phospholipid distribution in mice
Source: PLoS One. 2024 Nov 7;19(11):e0313205. doi: 10.1371/journal.pone.0313205 (PMC11542792; doi:10.1371/journal.pone.0313205)
Supplement: S6 Fig — Panel A, TG(17:0/18:1/18:2); B, TG(17:1/18:1/18:2); C, TG(16:0/17:0/18:1); D, Normalized expression of hacl1. These plots show the ratio of the experimental group divided by the control group, scaled by the error (standard deviation) of the measurements taken [20]. (DOCX) [file pone.0313205.s007.docx]

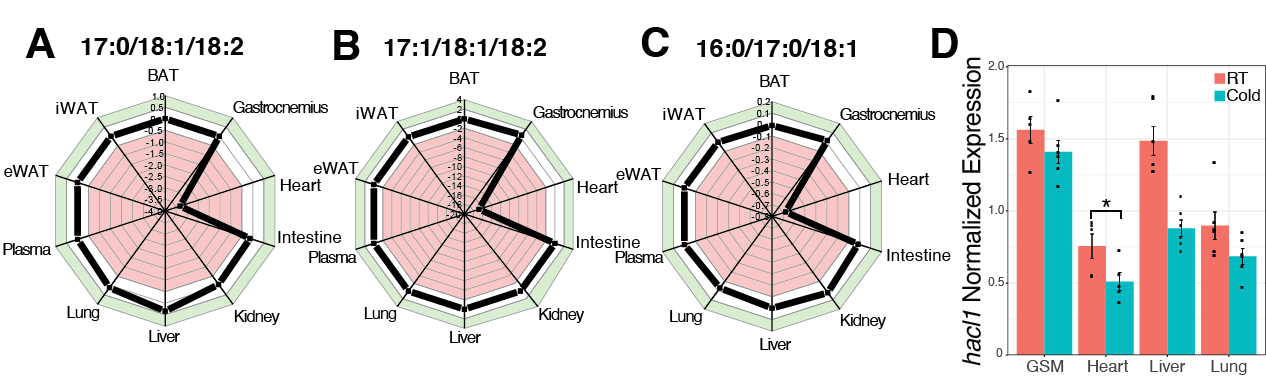


**Fig. S6. Error-normalised fold change plots of triglycerides comprising fatty acids expected to be shortened endogenously, by the product of *hacl1*, and expression of the gene.** Panel **A**, TG(17:0/18:1/18:2); **B**, TG(17:1/18:1/18:2); **C**, TG(16:0/17:0/18:1); **D**, Normalized expression of *hacl1*. These plots show the ratio of the experimental group divided by the control group, scaled by the error (standard deviation) of the measurements taken[1].
