# Supplementary material for: Systemic analysis shows that cold exposure modulates triglyceride accumulation and phospholipid distribution in mice
Source: PLoS One. 2024 Nov 7;19(11):e0313205. doi: 10.1371/journal.pone.0313205 (PMC11542792; doi:10.1371/journal.pone.0313205)
Supplement: S10 Fig — Top row, FA(22:5)-containing PCs; bottom row, FA(22:6)-containing PCs. These plots show the ratio of the experimental group divided by the control group, scaled by the error (standard deviation) of the measurements taken [20]. (DOCX) [file pone.0313205.s011.docx]

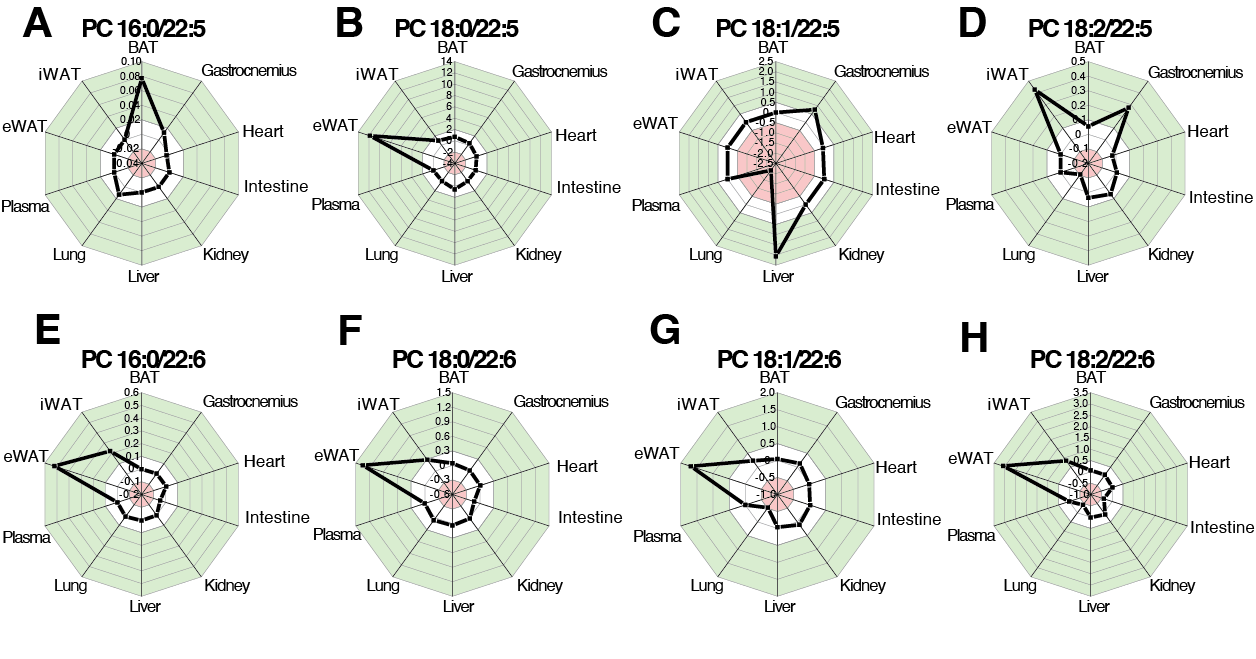


**Fig. S10. Error-normalised fold change plots of polyunsaturated-fatty-acid-containing phosphatidylcholines**. Top row, FA(22:5)-containing PCs; bottom row, FA(22:6)-containing PCs. These plots show the ratio of the experimental group divided by the control group, scaled by the error (standard deviation) of the measurements taken[1].

1. Furse S, Watkins AJ, Hojat N, Smith J, Williams HEL, Chiarugi D, et al. Lipid Traffic Analysis reveals the impact of high paternal carbohydrate intake on offsprings’ lipid metabolism. Communications Biology. 2021;4(1):163. doi: 10.1038/s42003-021-01686-1.
