# Supplementary material for: Systemic analysis shows that cold exposure modulates triglyceride accumulation and phospholipid distribution in mice
Source: PLoS One. 2024 Nov 7;19(11):e0313205. doi: 10.1371/journal.pone.0313205 (PMC11542792; doi:10.1371/journal.pone.0313205)
Supplement: S11 Fig — Panel A, Lipids from plasma; B, Lipids from the liver; C, Lipids from eWAT. (DOCX) [file pone.0313205.s012.docx]

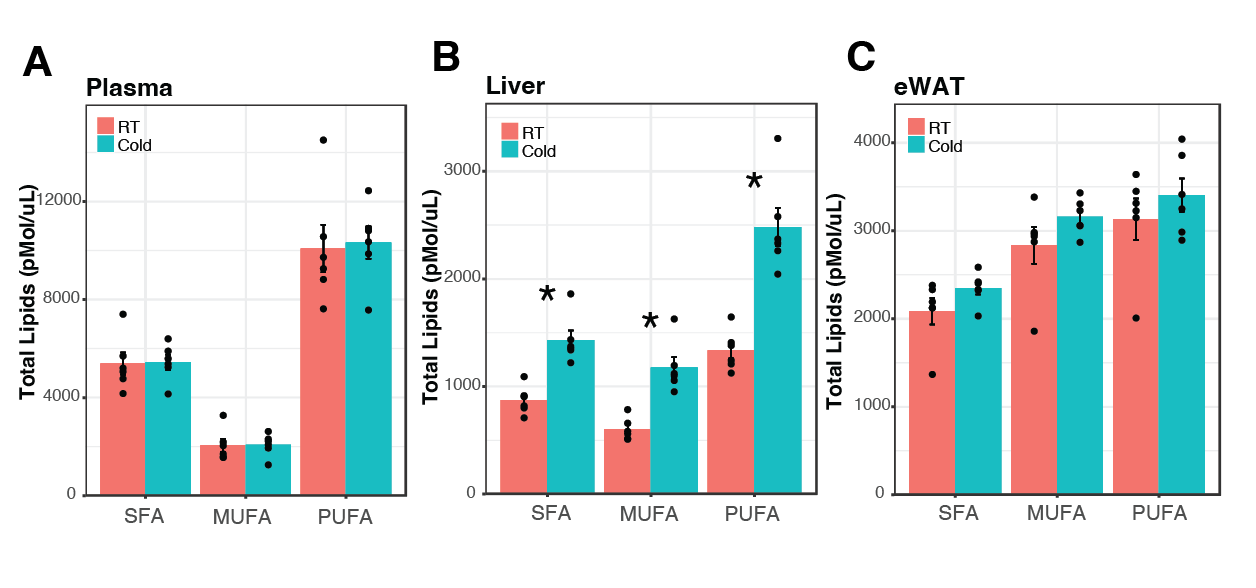


**Fig. S11. Concentration of fatty acids from all lipids, grouped into saturated (SFA), mono-unsaturated (MUFA) and poly-unsaturated (PUFA).** Panel **A**, Lipids from plasma; **B**, Lipids from the liver; **C,** Lipids from eWAT.
